# Theory-Driven Analysis of Natural Language Processing Measures of Thought Disorder Using Generative Language Modeling

**DOI:** 10.1016/j.bpsc.2023.05.005

**Published:** 2023-10

**Authors:** Isaac Fradkin, Matthew M. Nour, Raymond J. Dolan

**Affiliations:** a Max Planck University College London Centre for Computational Psychiatry and Ageing Research, London, United Kingdom; b Department of Psychiatry, University of Oxford, Oxford, United Kingdom; c Wellcome Trust Centre for Human Neuroimaging, University College London, London, United Kingdom; d State Key Laboratory of Cognitive Neuroscience and Learning, IDG/McGovern Institute for Brain Research, Beijing Normal University, Beijing, China

**Keywords:** Computational psychiatry, GPT-2, Natural language processing, Psychosis, Schizophrenia, Thought disorder

## Abstract

**Background:**

Natural language processing (NLP) holds promise to transform psychiatric research and practice. A pertinent example is the success of NLP in the automatic detection of speech disorganization in formal thought disorder (FTD). However, we lack an understanding of precisely what common NLP metrics measure and how they relate to theoretical accounts of FTD. We propose tackling these questions by using deep generative language models to simulate FTD-like narratives by perturbing computational parameters instantiating theory-based mechanisms of FTD.

**Methods:**

We simulated FTD-like narratives using Generative-Pretrained-Transformer-2 by either increasing word selection stochasticity or limiting the model’s memory span. We then examined the sensitivity of common NLP measures of derailment (semantic distance between consecutive words or sentences) and tangentiality (how quickly meaning drifts away from the topic) in detecting and dissociating the 2 underlying impairments.

**Results:**

Both parameters led to narratives characterized by greater semantic distance between consecutive sentences. Conversely, semantic distance between words was increased by increasing stochasticity, but decreased by limiting memory span. An NLP measure of tangentiality was uniquely predicted by limited memory span. The effects of limited memory span were nonmonotonic in that forgetting the global context resulted in sentences that were semantically closer to their local, intermediate context. Finally, different methods for encoding the meaning of sentences varied dramatically in performance.

**Conclusions:**

This work validates a simulation-based approach as a valuable tool for hypothesis generation and mechanistic analysis of NLP markers in psychiatry. To facilitate dissemination of this approach, we accompany the paper with a hands-on Python tutorial.

Psychiatric research has seen a surge in the use of natural language processing (NLP) methods for extracting clinically meaningful features from speech transcripts (e.g., clinical interviews) (1,2). Such features include both the content (3,4) and the form or organization of speech (5, 6, 7, 8, 9, 10, 11). Disruptions in the organization of speech, known as formal thought disorder (FTD), are particularly linked to psychotic disorders such as schizophrenia (12,13). Prototypical manifestations of FTD include a loosening of associative relationships between adjacent words or phrases [henceforth called derailment, as defined in (14)] and a tendency to drift away from the original focus of a narrative [henceforth called tangentiality, as defined in (14)].

Studies have shown that NLP methods can be used to capture such loosening of associations in patients’ speech (6, 7, 8,15,16), predict conversion to psychosis in at-risk populations (10,11), and contribute to identifying underlying neural mechanisms (17, 18, 19, 20, 21). Many of these studies have used semantic space models (e.g., latent semantic analysis, Word2Vec) (22, 23, 24) to quantify the semantic distance between words or phrases. These models represent individual words as vectors (i.e., word embeddings) in a multidimensional space trained (on large text corpora) to capture the statistical structure of natural language (see Figure 1 for a reduced, 2-dimensional illustration). Intuitively, FTD is predicted to result in greater distances among vector representations of words (Figure 1A, B) or sentences (Figure 1C–E) emitted during naturalistic speech.

**Figure 1:**
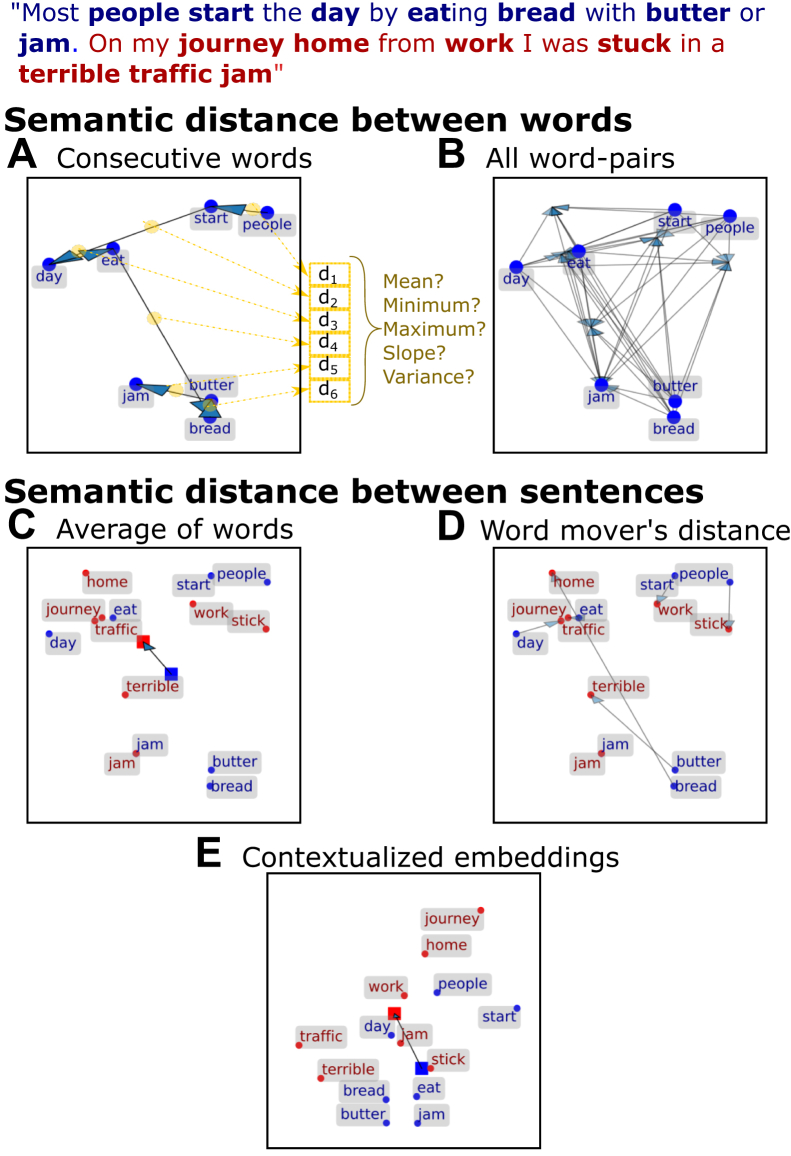
The representation of words in a semantic space and methods for calculating distance between words **(A, B)** and sentences **(C**–**E)**. Semantic space models yield straightforward embeddings for individual words, and the researcher is left to decide whether to calculate the distance between all words in a sentence **(B)**, or the entire narrative, rather than distances between consecutive words **(A)**. The computation of sentence-level semantic distances requires further analytic choices. The most common method generates a sentence vector as the mean of the (static) embedding vectors corresponding to each word in the sentence and then calculates the distance between these vectors **(C)**. More recently, a method quantifying the distance between sentences as the aggregate minimum amount of distance that each word in one group has to move to reach its closest word in the second group has been suggested **(D)**. Finally, methods relying on contextualized embeddings **(E)** account for how the same word can have a different meaning based on its context (e.g., the word jam in the current example). After deciding on the type of semantic distance, a researcher also must decide how to aggregate semantic distances across all word pairs or sentence pairs [see gold-colored illustration adjacent to **(A)**]. The semantic spaces depicted here correspond with a reduced, 2-dimensional representation (derived using principal component analysis) of popular semantic space models [GloVe in **(A**–**D)** and all-distilroberta-v1 in **(E)**].

Crucially, whereas previous studies have revealed the promise of NLP methods, they are nevertheless characterized by considerable heterogeneity in analytic pipelines and results (3,6,10,11,15,18,20,25, 26, 27, 28, 29, 30, 31, 32). For example, as shown in Figure 2, some studies have focused on semantic distances between words (see also Figure 1A, B), whereas others have focused on distances between sentences measured in a variety of ways (Figure 1C–E). Moreover, evidence for greater semantic distances in FTD has often been lacking, with some studies even showing an opposite effect (highlighted in Figure 2). These inconsistencies have rarely been scrutinized in a theory-driven manner, with greater attention being devoted to diagnostic or prognostic predictive accuracy, wherein the magnitude or even direction of effects receives little attention. Overall, this heterogeneity highlights the limits of our current understanding of what different NLP metrics actually measure.

**Figure 2:**
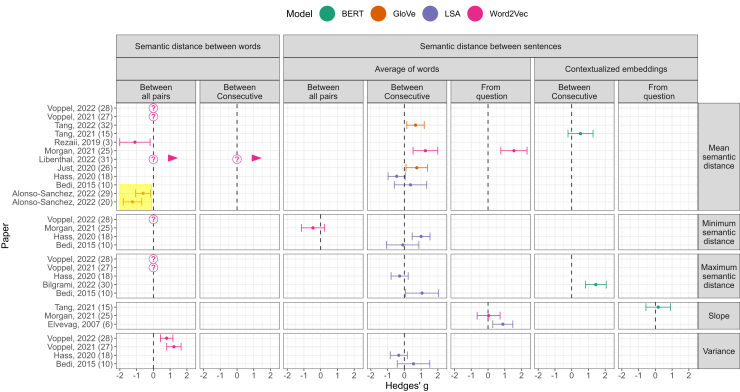
Methods and results of previous studies using semantic distance measures in formal thought disorder. Positive effect sizes denote greater (semantic distance) values in patients or high-risk individuals vs. control participants, in converters vs. nonconverters to psychosis, or a positive correlation with clinical formal thought disorder ratings. The top headings of each column show whether semantic distances were calculated between words or sentences and whether sentences were calculated by simply averaging the word embeddings included in that sentence or rather by using what is referred to as contextualized embeddings (Figure 1). The bottom heading shows whether distances were calculated between all pairs of words/sentences, consecutive pairs alone, or between a patient’s responses and an interviewer’s question. The heading on the right indicates how semantic distances were aggregated. The meaning of different types of semantic distance and their aggregation are illustrated in Figure 1. Question marks denote a metric that has been used but where the corresponding effect size was not reported (or could not be extracted; trends of an unclear effect size are denoted by small arrows). Additional details concerning the included studies and effect sizes are provided in the Supplement. Note that study (3) presented in the figure used a more complex measure (semantic density) that was simplified here for consistency with the other studies. BERT, bidirectional encoder representations from transformers; GloVe, global vectors for word representation; LSA, latent semantic analysis.

Much previous psychometric work in the field has focused on measuring correlations between NLP metrics and clinician-rated measures of FTD (30). Despite the value of this approach, it does not explain the success of NLP in capturing subtle linguistic markers of psychosis that are not readily identified by clinician-rated measures (15). Furthermore, this data-driven approach is limited in its ability to advance an understanding of how theoretical cognitive mechanisms of FTD manifest in altered NLP metrics. For example, one prominent theory suggests that FTD is caused by an impairment in maintaining global conversational context, thereby leading to excessive reliance on local context (33, 34, 35, 36). Whereas this theory may relate to aforementioned reports of smaller semantic distances between words in schizophrenia (Figure 2), these studies have often conflated local and global context by mixing proximal and distal word pairs (20,29) [but see (3)]. Furthermore, predictions relating to more complex, yet common, summary NLP metrics (e.g., the range or variance of semantic distances) (Figures 1 and 2) are even more difficult to make using intuition alone.

In computational psychiatry, theoretical predictions are usually evaluated by formalizing generative models, which are used to simulate data and generate quantitative predictions (37, 38, 39, 40). To date, notable attempts to simulate FTD have been informative but limited. For example, Hoffman *et al.* (41) examined predictions of different theory-based perturbations to an artificial neural network trained to generate stories, but these stories were markedly limited in structure, length, and lexicon (159 words). More recently, Bedi *et al.* (10) examined whether specific NLP metrics could recover disorganization generated by shuffling sentences in naturalistic texts. This manipulation is reminiscent of theories linking FTD to stochastic retrieval (42,43), but it remains incomplete because it only affects the order (but not the selection) of topics.

Here, we extended the work in these early reports by exploiting modern generative language models (e.g., generative pretrained transformer) (GPT) (44), which can generate human-like text (45,46) by optimizing next word prediction based on context. Whereas the architecture and training process of these models do not correspond with human language acquisition (47, 48, 49), recent studies have shown that the output (i.e., predictions) and internal representation of these models resemble some aspects of human linguistic processing (50, 51, 52), especially linguistic form (47). Crucially, regardless of how these models learn or represent language, the parameters that govern how text is generated (text-generation parameters) can be experimentally perturbed in multiple ways, some of which bear a resemblance to cognitive mechanisms previously hypothesized to underpin some aspects of FTD. This allows scrutiny of the construct validity of popular NLP metrics as their sensitivity to theory-based, a priori perturbations on realistic, human-like narratives.

We examined 2 text-generation parameters. First, as noted above, FTD has been proposed to reflect a specific impairment in the use of global linguistic context (33,34,53,54). This can be formalized by limiting the size of a memory buffer used to guide next-word selection. Given such limited memory span, the generated text is expected to lose the ability to maintain a single, coherent topic, yet maintain preserved (or even increased) local associations (33,34,53,54). In contrast, loosening of local (word-to-word) semantic associations has been repeatedly reported in schizophrenia, especially in more structured tasks (e.g., single-word associations, or category fluency tasks) (6,55,56). Thus, rather than a disruption in the balance between local and global context, FTD can result from a generalized impairment in using (any) context to constrain word selection. This may reflect abnormalities in semantic representation (i.e., over-inclusive semantic networks) (42,57,58) or more noisy retrieval from (intact) semantic memory (56,59, 60, 61). For simplicity (and without taking sides on the representation vs. access debate), we formalized such generalized underconstraint by increasing the temperature (i.e., stochasticity) of word selection.

We do not argue that these 2 text-generation parameters represent an exhaustive set of the mechanisms that are at play in FTD (e.g., they are not designed to capture phenomena such as perseverative speech, neologisms, echolalia), nor do we argue that their theoretical plausibility implies (or depends upon) any biological plausibility to the transformer architecture itself. Instead, we view GPT solely as a tool for simulating realistic narratives that can be perturbed using theoretically informed parameters, which may reflect a variety of biologically plausible mechanisms [e.g., limited contextual span may result from NMDA hypofunction (62,63), whereas greater stochasticity may reflect synaptic disconnection (42)]. A key motivation is to use this simulation-based method to scrutinize the construct validity and failure modes of popular NLP metrics and to evaluate the predictions of these mechanisms in relation to previous NLP findings. To further encourage extending this approach to additional theories and future metrics, we complemented the paper with a hands-on tutorial for using free out-of-the-box tools for natural text generation (64).

## Methods and Materials

### Simulating Narratives

**Figure 3:**
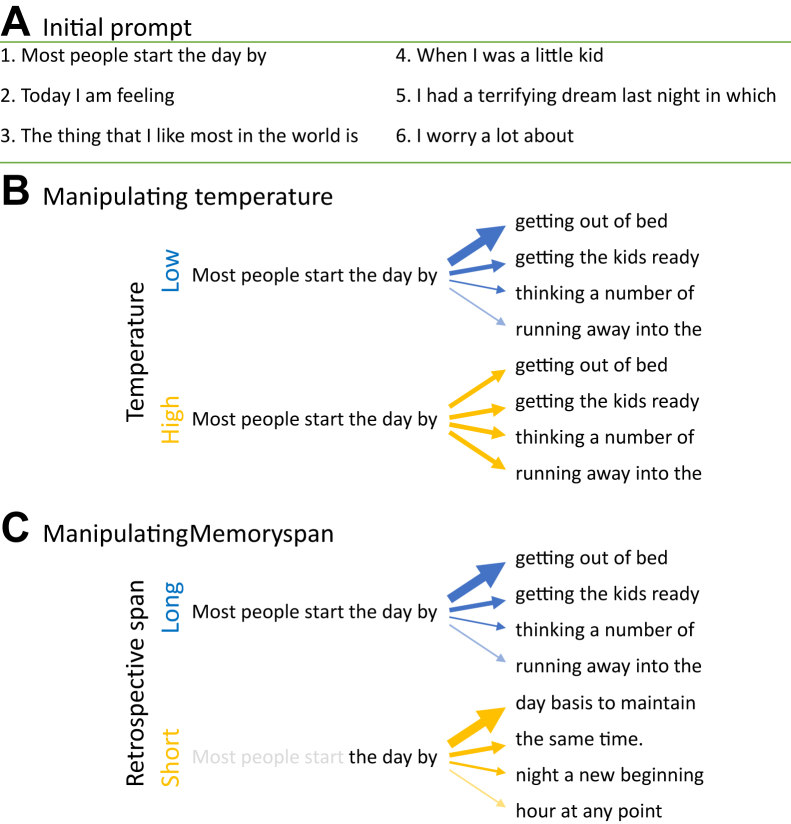
Key text-generation parameters controlling the generation of simulated free narratives. Each narrative was started by providing an initial prompt **(A)**. Narratives potentially mimicking those found in formal thought disorder can be generated either by increasing the temperature (stochasticity) of the sampling [temperature in **(B)**] or by limiting the model’s ability to “remember” the global context [memory span in **(C)**].

We simulated narratives using GPT-2 (44,65), a transformer-based artificial neural network, where the input is a body of text (i.e., prompt), and the output is a probability distribution over tokens (i.e., words, subwords, and punctuation marks) used to sample the next token. Each narrative starts with 1 of 6 brief conversational prompts (Figure 3A), and the model iteratively generates narratives consisting of 200 tokens.

We modulated 2 key text-generation parameters. First, underconstrained word selection was formulized by increasing the temperature parameter (Figure 3B). Second, a limitation in using global context to guide next-word selection was formulized by reducing the span of the (memory) context presented to the model as a prompt at each time step (Figure 3C). For each prompt, we generated 200 narratives using temperature parameters in the range 1 to 5 (fixing the memory span to 200) and an additional 200 narratives using memory span in the range 3 to 200 (fixing the temperature to 1).

We implemented several conventions to encourage GPT to produce more realistic text. First, tokens were generated using beam-search sampling (66), wherein, for each iteration, the model generated 5 potential 3-token trajectories, choosing the next word based on the joint probability of the entire trajectory (see Figures S1–S3 for sensitivity analysis). Second, for each sampling step, we excluded the 1% of words with the lowest probability estimates (nucleus sampling) (66). Third, to minimize repetitiveness, we prohibited the model from repeating the same pair of words (e.g., 2-grams). Finally, we prohibited the model from generating some internet-based tokens (e.g., “https://,” new-line characters).

### Clinical Ratings of Simulated Narratives

We examined the face validity of the simulated narratives in terms of how well they mimicked some aspects of FTD. For this purpose, a subset of 249 narratives were rated by 2 clinicians experienced in clinical assessment of patients with psychosis (IF and MMN, who were blind to the perturbation governing each narrative) using the Thought and Language Disorder Scale (67). Given the nature of perturbations and narratives, these ratings were restricted to specific dimensions of disorganized speech (i.e., positive FTD): derailment (measuring loose associations between adjacent phrases), dissociation (measuring a complete lack of associations between adjacent sentences or words), and tangentiality (measuring how quickly a text deviates from initial meaning) (see the Supplement for interrater reliability and additional details).

### NLP Measures of Semantic Distance

We analyzed simulated narratives using common NLP measures of semantic distance. First, we operationalized derailment as greater cosine distance (1-cosine similarity) between the vector embeddings of consecutive words or sentences (Figure 1). Then, to obtain a single derailment metric for each narrative, we followed a convention used in previous studies by calculating either the mean, minimum, maximum, or variance of these distance measures (Figure 1). Second, we operationalized tangentiality as the average rate (i.e., slope) at which the semantic meaning of a sentence diverged from the initial prompt.

In our analyses, we focused on both the direction and size of the effects (measured using Spearman correlations) and their consistency across prompts and models. Statistical significance was not examined because it depends heavily on the number of simulated narratives.

Word-level embeddings were extracted from 3 popular semantic space models: Word2Vec (23), GloVe (22), and fastText (68). We examined 2 approaches for computing sentence-level metrics from these word-level embedding models. First, we encoded a sentence as the mean of the vectors corresponding to each word in the sentence and then calculated the cosine distance between such averaged vectors (Figure 1C) (10,18,32,69). Second, we used the Word Mover’s distance (70,71) metric, which quantifies the aggregate minimum amount of distance that each word in 1 sentence has to “move” to reach its closest word in the second sentence (Figure 1D). To ensure that these measures focused on semantics rather than simple repetition, words appearing in both sentences were excluded prior to distance calculations (the results of relaxing this constraint are presented in Figures S4 and S5).

It should be noted that the above measures of semantic distance fail to account for how the same word can have a different meaning based on its context. Thus, we also calculated semantic distance between sentences using more sophisticated contextualized sentence embedding models (namely, all-MiniLM-L12-v2; all-mpnet-base-v2; all-distilroberta-v1) (72), which excel at representing the role of each word in its context (e.g., the word “jam” in Figure 1E).

Prior to calculation of the above NLP measures, narratives were preprocessed according to conventional practices. First, simulated narratives were tokenized into sentences (based on full stops), and common contractions were expanded (e.g., “wouldn’t” was changed to “would not”). These sentences were used for analyses based on contextualized embedding models. Analyses based on static embedding models were preceded by the tokenization into words; the removal of stop words (e.g., determiners, coordinating conjunctions, prepositions), single letters, and non–alpha-numeric characters; and the conversion of the remaining words into their dictionary form (i.e., lemmatization; e.g., “going” changed to “go”).

## Results

### Perturbations to GPT2 Text-Generation Parameters Mimic Some Aspects of FTD

Perturbation to both temperature and memory span led to less coherent narratives - reminiscent of clinical presentations of FTD (Table 1). This lack of coherence was supported by clinical ratings indicating that both perturbations increased derailment (temperature: *r*_*s*_ = 0.53, *p* < .001; memory span: *r*_*s*_ = 0.32, *p* < .001; *p*_*difference*_ = .10), and dissociation of thinking (temperature: *r*_*s*_ = 0.52, *p* < .001; memory span: *r*_*s*_ = 0.66, *p* < .001; *p*_*difference*_ = .21). Furthermore, whereas tangentiality was correlated with both parameters (temperature: *r*_*s*_ = 0.37, *p* < .001; memory span: *r*_*s*_ = 0.68, *p* < .001), the correlation with memory span was significantly larger (*p*_*difference*_ = .012). Importantly, given that GPT is trained on web-based language, some narratives (19.27%) were more similar to written, web-based text than to disorganized speech (see the Supplement). However, although such narratives diverge in content from what a patient might say, they were not significantly different from speech-like narratives in the correlations with form-based clinical ratings (*p*s > .189). Overall, these findings suggest that both parameters of interest covary with some clinically meaningful aspects of FTD. Next, we turn to the main aim of the paper, namely examining the theoretical underpinning and construct validity of popular NLP metrics of FTD.

**Table 1:** Illustrating the Effect of Potential Mechanisms of Formal Thought Disorder

Condition	Quote
Baseline	Most people start the day by getting up and going to work, and then they don’t have time to do anything else. But if you have a lot of time, you can do other things. You can go to the gym, or go for a walk. If you’re not in the office, then you could do something else, like go out and play with your kids. It’s not like you need to be at work every day. I think that’s one of the things that really makes it work for me. When I’m at home, I can spend time with my family. That’s a big part of my life. And then I have the time in between to just do whatever I want. So it’s nice to have that flexibility. For me, it works out really well. Not having to worry about work is a huge benefit. There’s no pressure to get up at the crack of dawn. No one’s trying to wake you up.
Increased Temperature	Most people start the day by reading some of those old books you brought home in the past, they’re still there. It is the way our ancestors always taught us a way you have to go in order get a good understanding of the human mind, the universe. So the next day, you do not do it the same again. In fact your mental life, as is true of our physical bodies we have our mind as we do, a kind of “second body,” one of several different kinds. If we can use it to learn, we might do more for ourselves then the old habits were able to provide. As we learn our ways and we use our new body to make it more productive; we don’t lose things. You lose them to other people to use. They come back. Our bodies may grow in different proportions. And they may also have other qualities as well, maybe some different reactions to whatever happens, but not necessarily our body will feel or look “the same.”
Decreased Memory Span	Most people start the day by getting up and going to the bathroom. I didn’t want to be alone, so I thought I’d give it a shot. I don’t know if it’s true or not, but I don’t think it’s a good idea to make sure that you have a good understanding of how to use it. I don’t know if it’s because I’m old or because it’s a good idea to do so. If you’re looking for a way to get the most out of it. I’ve been doing this for a long time, and I think it’s time for a change. I’m going to go back to my room and go to sleep. I was able to get a good night’s sleep. It’s a great way to get a good night’s sleep. It’s not that I don’t want to do it. I’m not going to sit here and tell you that I don’t think you’re going to get away with that.

### The Effects of GPT2 Word-Selection Temperature on NLP Metrics

**Figure 4:**
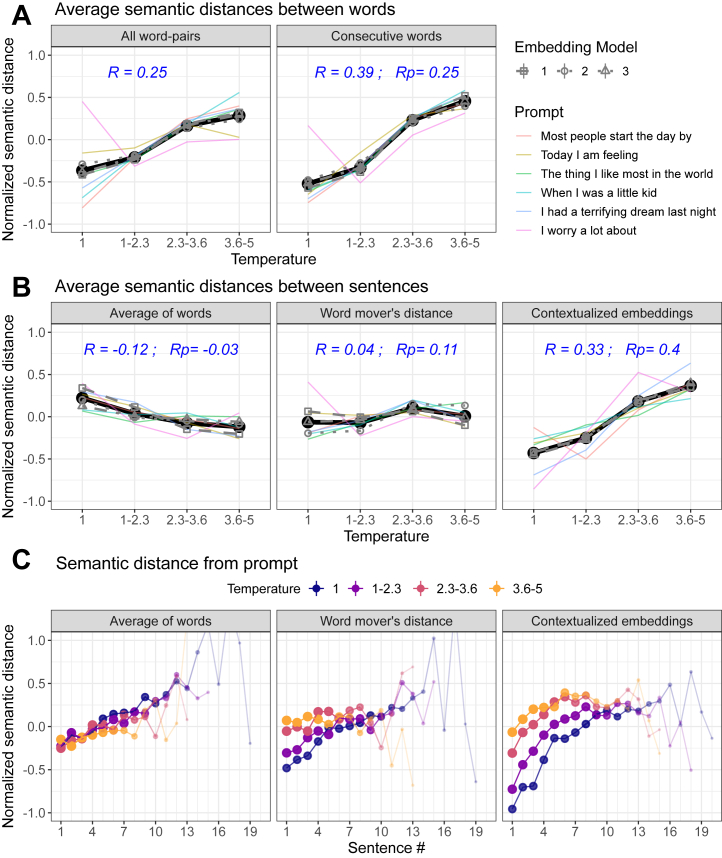
Effects of temperature manipulations on semantic distance measures of derailment **(A, B)** and tangentiality **(C)**. Overall effect sizes were calculated as the average Spearman correlations between (nonbinned) temperature and the respective semantic distance (R), with a potential control for the average number of words in a sentence (Rp). The consistency of these effects among prompts (averaged across models) and models (averaged across prompts) is represented by colors and line types, respectively (static embeddings are 1: fastText, 2: GloVe, 3: Word2Vec; contextualized embeddings are 1: all-distilroberta-v1, 2: all-MiniLM-L12-v2, 3: all-mpnet-base-v2). Smaller points and thinner lines in **(C)** denote rare sentence numbers in the respective condition.

As expected, increasing temperature increased the semantic distance between words (Figure 4A). We predicted a similar positive correlation between temperature and semantic distance measured between adjacent sentences. Empirically, however, the sentence-level results varied in both magnitude and direction depending on how sentences were encoded (Figure 4B). More specifically, we found the expected positive correlation when using contextualized embeddings, wherein sentence meaning accounted for the relationships between words within a sentence. Conversely, aggregating the distances between individual words produced a much weaker effect (word mover’s distance in Figure 4B). Furthermore, this relationship was reversed when measuring distance between consecutive sentences as a simple average of (static) word embeddings (average of words in Figure 4B).

A follow-up analysis suggested that the latter, surprising negative relationship was mediated by the effect of temperature on increasing sentence length (*r* = 0.69). Thus, whereas a sentence consists of words expressing a complete thought, increased temperature renders such coherent semantic units harder to enclose (Figure S6). As sentence length increases, the averaged embedding vector is expected to approach the zero vector because the orientations of individual word embeddings cancel each other out (especially under high temperature). Consistent with this conjecture, controlling for the average number of words per sentence (i.e., using the residuals of temperature after regressing it on the average number of words) weakened the negative effect of temperature on the semantic distance between averaged vectors (see average number of words in a sentence in Figure 4B). Thus, whereas the averaging of static word embeddings is the most common approach for calculating semantic distance between sentences (Figure 1), it fails to reveal an expected effect of temperature and was strongly influenced by confounds such as sentence length.

The autoregressive nature of GPT means that higher temperature does lead not only to the selection of less constrained words but also to the formation of a less constraining context. Thus, the weight of the original prompt on the evolving context will gradually decrease, potentially resulting in tangentiality. Crucially, however, although increasing the temperature increased the distance between the prompt and the first sentence, it tended to decrease rather than increase the slope of the divergence of subsequent sentences, most likely reflecting a ceiling effect (Figure 4C; see also Figures S7 and S8 for additional demonstrations).

### The Effects of Limited Memory Span on NLP Metrics

**Figure 5:**
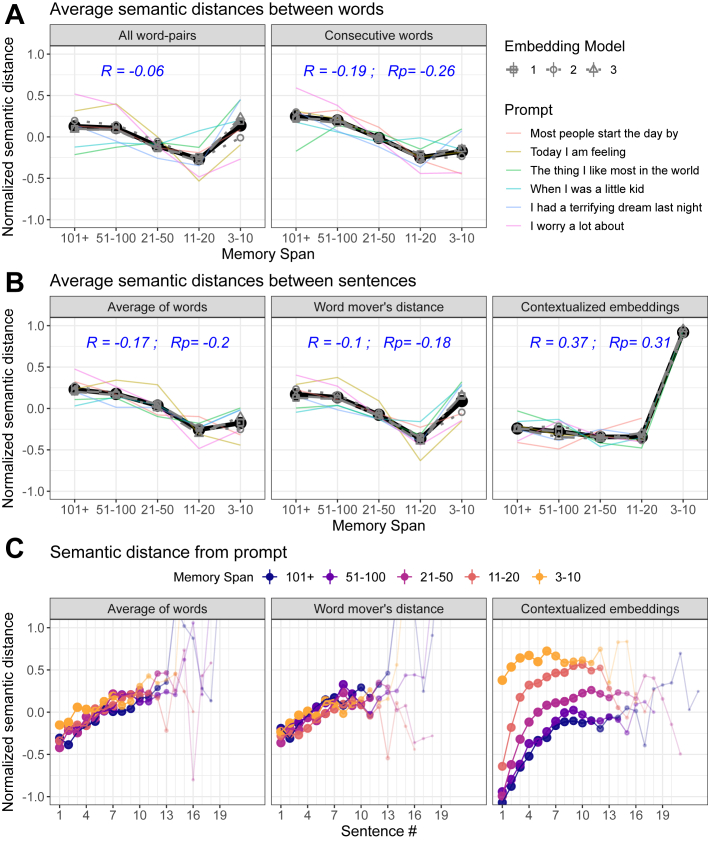
Effects of manipulating memory span on semantic distance measures of derailment (**A**, **B**) and tangentiality (**C**). Overall effect sizes were calculated as the average Spearman correlations between (nonbinned) memory span and the respective semantic distance (R), with a potential control for the average number of words in a sentence (Rp). The memory span variable was reversed prior to calculating these correlations because (opposite to temperature) formal thought disorder is linked to lower memory span. Accordingly, the memory span axes are presented here in reverse (i.e., decreasing) order. See the caption of Figure 4 for further details.

As predicted, a limited memory span increased the semantic distance between consecutive sentences encoded using contextualized embeddings (Figure 5B). Conversely, and in stark contrast to the effects of temperature, decreasing memory span did not consistently increase semantic distance between words (Figure 5A). Instead, at least in the case of consecutive words, semantic distances were reduced. These results confirm an intuitive hypothesis that a limited memory span shifts the balance between global and local context such that a word is sampled mostly based on the local context preceding it, and previous sentences are disregarded.

Critically, our findings extend upon this intuitive prediction by revealing a pattern of nonmonotonic effects that vary across metrics (Figure 5B). Thus, an increased semantic distance between sentences was evident when memory span was low enough to exclude words of a preceding sentence (i.e., span of 3–10 words). Conversely, a transition from high (e.g., 100+) to intermediate (e.g., 11–20) levels of memory span decreased semantic distance between sentences, reflecting the fact that such intermediate levels entail that each sentence is only determined by the preceding sentence (and thus closely relates to it). This nonmonotonicity means that if, for example, patients vary in the extent of memory span impairment, different cohorts (and different patients within a cohort) may exhibit different (potentially opposing) results. It should also be noted that the specific pattern of results varies between contextualized and static embedding models such that the transition from maximal to minimal memory span increases semantic distance only in the former.

Examining our NLP measure of tangentiality revealed that in contrast to temperature manipulations, reducing memory span did increase the rate of the divergence of sentences from the prompt. As shown in Figure 5C, this effect is most evident for the fourth sentence onward (see also Figure S7), which tends to be, on average, approximately 50 words away from the prompt (assuming an average of 14 words per sentence). Notably, however, memory span also had a strong effect on the semantic distance between the prompt and the first sentence, which also led to a ceiling effect similar to the one that was reported for the temperature manipulations described above. This ceiling effect was most evident for very low memory span (i.e., 3–10) and led to nonmonotonicity (Figure S8) that diverged from the one that was reported for NLP metrics of derailment (where the effect was maximal for a memory span of 3–10).

### The Importance of Other Analytic Choices

Our findings suggest that sensitivity for recovering and dissociating the computational parameters that we examined here is optimized by combining the semantic distances between consecutive words and the semantic distances between sentences (encoded using contextualized embeddings). Of course, researchers have a variety of other choices. Reassuringly, we found that the choice of which specific static or contextual embedding model to use had minimal effect (compare line types in Figures 4 and 5). Conversely, the results varied to some extent among conversational prompts (compare colors in Figures 4 and 5). Whereas we found no evidence that specific prompts or prompt types (e.g., negative vs. neutral) were consistently advantageous (Figure S9), this result suggests that researchers should optimally examine the generalizability of their results across prompts.

**Figure 6:**
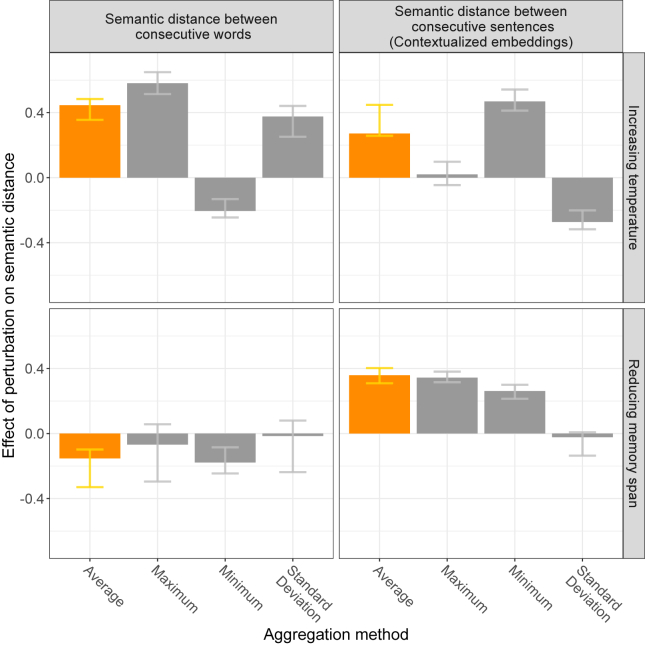
Sensitivity of different methods (x-axis) for summarizing semantic distances within a narrative for the 2 types of perturbation. Error bars represent consistency across embedding models and probes, calculated here as the interquartile range. The results of using the average for aggregation summarize the respective effects presented in Figures 4 and 5.

Finally, whereas for the above results we calculated narrative-level derailment by averaging semantic distances across all word pairs or sentence pairs, previous studies have used a variety of alternative aggregation methods, focusing on variability or extreme semantic distances (Figure 1). Critically, as shown in Figure 6, the benefit of using such alternative methods has been small and inconsistent. These results suggest that researchers may prefer to focus on the average (NLP-measured) derailment of narratives or otherwise choose an aggregation method based on the hypothesized mechanism and measure of interest.

## Discussion

Here, we argue for the value of a simulation-based approach for improving the theoretical foundations of NLP-based analyses in psychiatry. To illustrate the general approach, we first demonstrated the capacity of generative language models to generate realistic text that mimics aspects of FTD through parametric tuning of cognitively meaningful parameters. Next, we showed how these hypothesized parameters make different predictions regarding the associative structure of the generated text. Finally, and most importantly, we demonstrated marked variability in the ability of common automated NLP metrics to capture these predictions, thereby providing a toolkit that we hope will improve the rigor of this burgeoning research field in the future.

In our simulations, we focused on 2 parameters: a) higher temperature (stochasticity) in word selection reflecting a generalized impairment in using context to constrain word selection, and b) limited memory span reflecting a specific impairment in using global context. We found that both parameters could explain weakened associations among consecutive sentences, whereas associations among words were weakened by higher temperature but strengthened by limited memory span. These unique predictions of limited memory span are broadly consistent with previous findings, in particular with evidence for decreased semantic distances between single words in FTD (Figure 1). Interestingly, whereas this result in isolation can also be assumed to reflect negative FTD symptoms such as perseveration (20,29), this alternative explanation also predicts smaller distances between sentences (30), which has not been reported in previous studies (Figure 1).

A key contribution of a simulation-based approach arises out of an ability to quantitatively compare the sensitivity of different NLP metrics. Indeed, we found that impairments in using local and global context were best captured by measuring semantic distances between consecutive words and between contextualized sentence embeddings, respectively. Strikingly, common approaches such as measuring the distances between all words in a narrative (20,29) or representing sentences by averaging (static) word embeddings (10,18,32,69) were noisy, confounded, and less able to dissociate the 2 parameters. Another key finding is that the effects of limited memory span on NLP-measured derailment and tangentiality were not monotonic. For example, forgetting the last sentence reduced its influence on subsequent word selection, whereas forgetting the broader context may in fact increase the influence of this last sentence. This nonmonotonicity means that patients with different levels of global context insensitivity may show opposite effects, thereby complicating the interpretation of group effects. Finally, whereas previous studies attempted to capture more complex dynamics of speech disorganization by accounting for how semantic distances vary within a narrative, our simulations showed that these alternative metrics are, in most cases, less sensitive than simple averaging. Overall, our results demonstrate the contribution of a simulation-based approach to interpreting heterogeneity in previous findings and guiding the selection of theoretically informed metrics in future studies.

An important question for future studies concerns the interaction between a generalized underconstraint (42,43) and an impairment in maintaining global context (33,34,54,73,74). Interestingly, in a recent paper, it was hypothesized that a repeated difficulty in maintaining global context (or intent) may lead to overly inclusive semantic networks through excessive adaptation of semantic representations (57). Such a combined mechanism adds another layer of complexity to interpreting NLP metrics in which the 2 reported mechanisms, with partially opposing effects, may operate in tandem (Figure S10). One pathway for examining this question would involve developing efficient methods to fit text-generation parameters directly to clinical transcripts.

We acknowledge several limitations of our paper. First, modern language models are dissimilar to human language not only in their architecture but also in their training corpus. Indeed, about one-fifth of the clinically rated narratives were more similar to blogs or news reports than to natural speech. Importantly, however, perturbations had a similar effect on speech-like and non–speech-like narratives.

Second, language models, and thus their suitability for simulating FTD, are limited by well-known cultural biases (75). Of course, this problem also affects common NLP metrics, which were recently shown to have limited generalizability even across common languages (69). Third, FTD is susceptible to the influence of affective and interpersonal factors (76, 77, 78, 79, 80), emphasizing another key limitation of language models—that they are trained on form and are not grounded in the real world (48,49). Interestingly, the ability to simulate perturbed narratives at scale (for any possible affective or neutral prompt, with increasing support for different languages) paves the way for systematically testing how content (e.g., valence), language, and other variables moderate the sensitivity of NLP-based metrics (Figure S9).

Finally, we did not examine the effects of the simulated parameters on NLP measures that do not focus on semantic distance (e.g., speech-graph analysis, referential cohesion) (16,81). However, the parameters we examined here may affect these and other metrics, as indicated, for example, by our secondary finding that higher temperature predicted longer sentences. Longer sentences may be reminiscent of the pressured speech seen in some manifestations of schizophrenia and in the manic phase of bipolar disorder.

The limitations that we have outlined show that despite the promise of large language models, currently, they do not offer a comprehensive, biologically plausible account of human language or its disruptions (49). Nonetheless, as we demonstrated here, the flexible and realistic nature of the output of these models can help develop a more theoretically and psychometrically informed NLP approach to FTD and guide future hypothesis generation. Indeed, an ability to ascribe mechanisms to specific NLP metrics paves the way for better linkage of FTD to other symptom dimensions, cognitive phenomena, and even drug effects (82). For example, NLP predictions of our temperature parameter may be linked to disorganized behavior and computational indices of choice stochasticity in decision making (83,84), whereas the predictions of limited memory span may be correlated with measures of working memory and attractor instability (85,86).
