# Supplementary material for: Theory-Driven Analysis of Natural Language Processing Measures of Thought Disorder Using Generative Language Modeling
Source: Biol Psychiatry Cogn Neurosci Neuroimaging. 2023 Oct;8(10):1013–23. doi: 10.1016/j.bpsc.2023.05.005 (PMC11932972; doi:10.1016/j.bpsc.2023.05.005)
Supplement: Supplementary Material [file mmc1.pdf]

# SUPPLEMENTARY INFORMATION

# Theory Driven Analysis of Natural Language Processing Measures of Thought Disorder Using Generative Language Modeling

Fradkin *et al.*

Code and text-generation tutorial are available at https://osf.io/xhefa/

**Details concerning the method used to generate Figure 1, providing a selective review of previous findings of NLP in FTD.**

Studies included in Figure 1 were located using a combination of Google scholar search (combining search terms like "natural language processing" & "semantic coherence" with search terms like "thought disorder" and "schizophrenia"), a snowball approach using the references of existing papers or previous reviews (1, 2). Only published papers that included at least one measure of semantic distance were included. We included papers comparing patients or CHR individuals to controls (3–11), papers investigating conversion to psychosis among CHR individuals (12–14), papers examining the correlations between NLP metrics and clinician-rated measures of FTD (11, 15, 16), and one paper examining correlations between NLP metrics and social functioning (17).

Whereas most studies reported on 'semantic coherence' as measured by cosine similarity, we reversed all effects in our figure to match them with the measures of semantic distance (1-semantic coherence) we analyzed in our paper. For papers that did not report sufficient statistics to calculate an effect size, we relied either on published data (14), or on digitization of figures to estimate individual data points (12). In cases in which these methods were also unavailable we report the main method used in the study but not the effect size (in cases in which a trend is evident from the data we report its direction but not size or significance).

**Sensitivity of the simulation results to different beam-search trajectory horizons**

Here we examine whether the results reported in the paper were sensitive to other values of beam-search trajectory horizons (i.e., how many words the model plans and generated on each iteration). Comparing Figure S1 to Figure 4, and Figure S2 to Figure 5 shows that the results are very consistent, with very few differences.

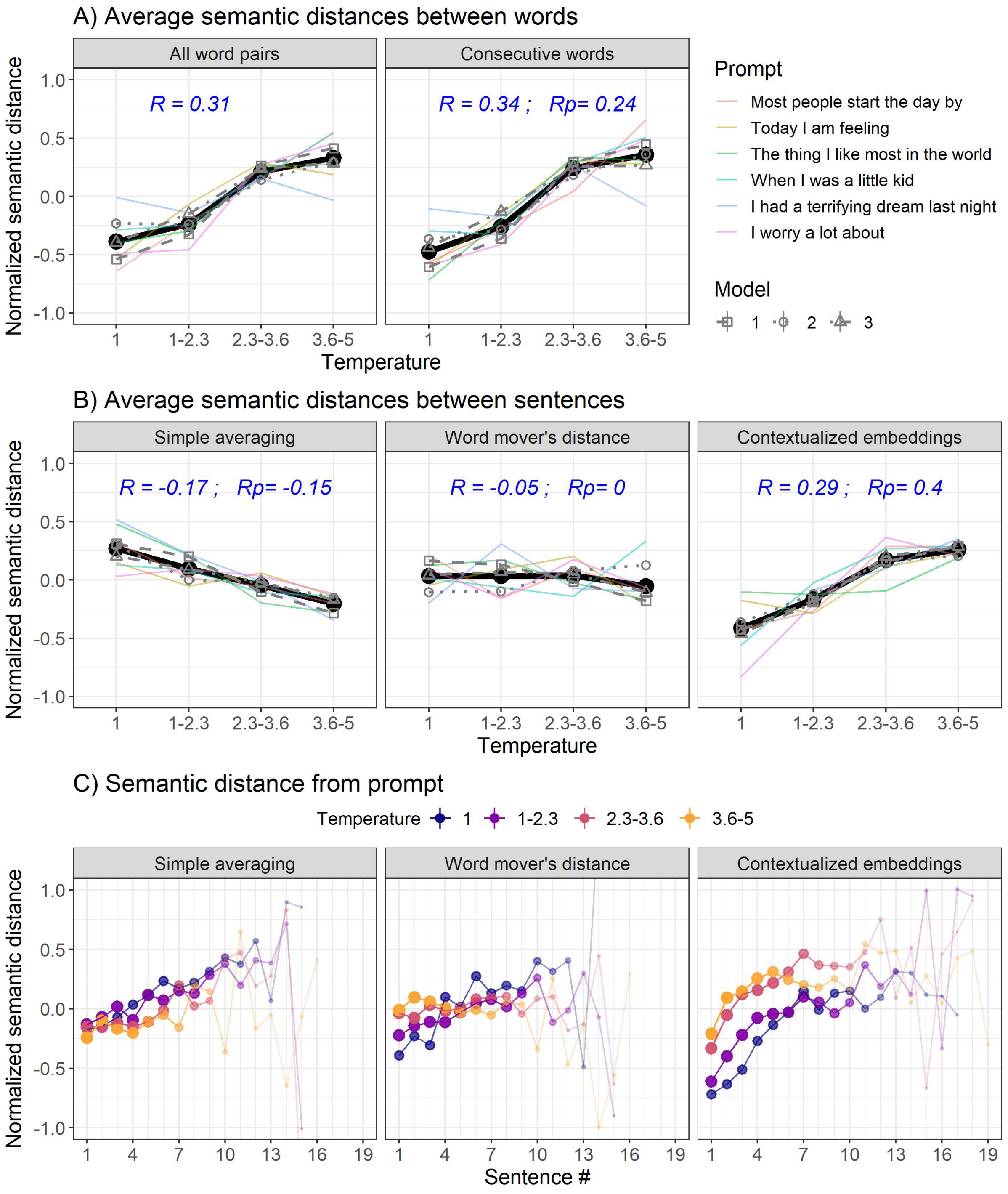


*Figure S1 - Effects of manipulating temperature on semantic distance measures of derailment (A and B) and tangentiality (C), for a beam-search trajectory horizon of 10 tokens (instead of 3). See the caption of Figure 4 for further details.*

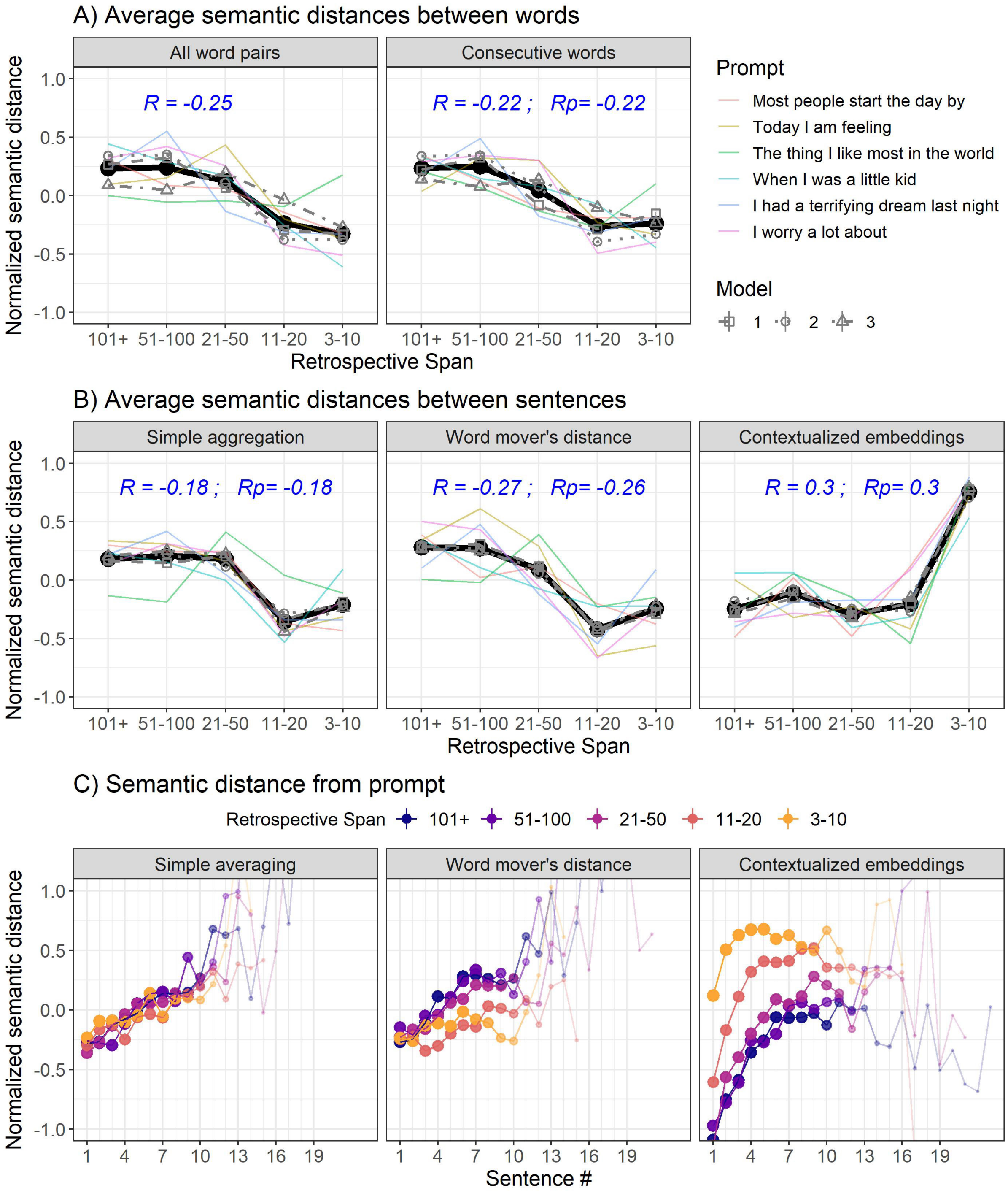


*Figure S2 - Effects of manipulating retrospective span on semantic distance measures of derailment (A and B) and tangentiality (C), for a beam-search trajectory horizon of 10 tokens (instead of 3). See the caption of Figure 4 for further details. Retrospective span axes are presented in reversed (i.e., decreasing) order since (opposite to temperature) formal thought disorder is linked to lower retrospective span*

## The effects of beam-search trajectory horizons on semantic distance

Here we examined whether reducing beam search trajectory horizon (while keeping temperature and retrospective span at their optimal levels) can also produce FTD-like narratives, as measured by different semantic distance measures. Indeed, this variable can be considered as a manipulation of 'prospective memory span', or the degree of discourse planning. The results, depicted in Figure S3 suggest a weak effect, such that lower prospective span slightly reduced between-sentence coherence (measured as contextualized embeddings). In addition to being very weak, this effect was not very stable across prompts, nor did it affect distances between groups of N-words. However, these 'null results' should be interpreted with caution because we examined a relatively small range of prospective span values, and we did not examine whether the effect strengthens when >5 beams are used.

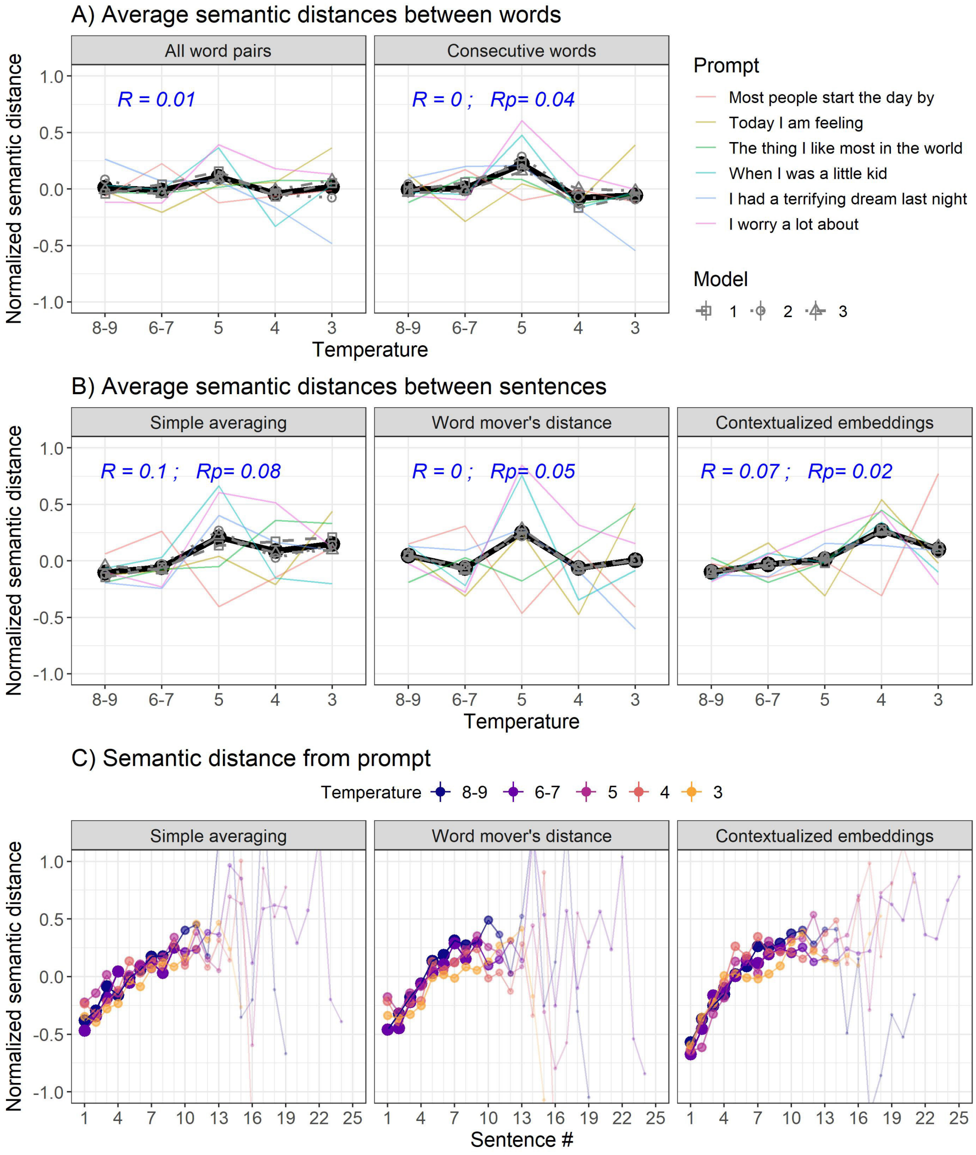


*Figure S3 - Effects of manipulating beam-search trajectory horizon (prospective span) on semantic distance measures of derailment (A and B) and tangentiality (C). See the caption of Figure 3 for further details. Prospective span axes are presented in reversed (i.e., decreasing) order since (opposite to temperature) formal thought disorder is linked to lower prospective span*

**Additional details regarding the clinical ratings procedure**

A random subset of 249 narratives (25 for each of 5 ranges of memory span, namely: <10, 11-20, 21-50, 51-100, 101-200, and 31 for each of 4 ranges of temperature, namely: 1, 1-2.3, 2.3-3.6, 3.6-5), were rated by two raters (I.F. and M.M.N.), experienced in clinical assessment of patients with psychosis, using the Thought and Language Disorder Scale (TALD)(18). The raters were aware of the fact that the narratives were generated by GPT but were blind to the level and type of perturbation of each narrative.

We focused on dimensions of positive FTD (thus, excluding negative FTD dimensions like poverty of thought, blocking etc.) that do not depend on a conversational setting (excluding dimensions like crosstalk, logorrhoea, etc.), idiosyncractic language (excluding symptoms like neologisms, manneristic speech, paraphrasia, etc), or sound-based speech (excluding symptoms like clanging, echolalia, etc.). We also did not examine less-specific dimensions like poverty of content of speech, reflecting general vagueness, since whether it relates more to negative (19) or positive (18, 20) FTD symptoms remains unclear. Thus, we focused on 3 main dimensions: Derailment (characterized by local associations that are clearly but obliquely or vaguely related), Dissociation of thinking (characterized by completely unrelated connections, in contrast to derailment), and Tangentiality (characterized by a global structure wherein content drifts away from where it originally started). Whereas these three dimensions have a clear theoretical link with the perturbed parameters, we also explored whether our perturbation might affect Circumstantiality (characterized by circuitous, long-winded speech, wherein the main point gets lost in overly specific or repetitive detail, without losing the original topic completely) or Perseveration (characterized by repetitive text).

Inter-rater reliability ratings were satisfactory for derailment ($\kappa = .48$), dissociation ($\kappa = .74$), and tangentiality ($\kappa = .57$), especially when considering the fact that narratives were relatively short, and did not include back-and-forth type conversations that characterize an actual clinical interview. Inter-rater reliability for the average of these three dimensions was also satisfactory (ICC = 0.70). Conversely, the reliability of circumstantiality ($\kappa = .11$) and perseveration ratings ($\kappa = .11$) was insufficient, further highlighting the aspects of FTD that cannot be reliably explained by the examined perturbations (i.e., the lack of reliability is explained by very low 'true variance'), and leading us to focus only on the former three dimensions in the results reported in the paper.

Finally, each narrative was also rated with respect to how `speech-like` it is. As reported in the main text, 19.27% were more similar to web-page written language (e.g., "The thing I like most in the world is a quiet moment of reflection,' says Larkin, 34, the president of Davenport Associates, a consulting agency in Washington DC that helps foreign businesses and companies set aside assets' "). An additional 24.50% were characterized by unsuitable use of third-person pronouns, but otherwise were sufficiently similar to human speech (e.g., "I had a terrifying dream last night in which I saw a man in a black suit and a white shirt, and he said, 'I'm going to kill you. I'm coming for you.' And I said to myself, 'this is not a joke. This is serious. And that's when I realized that I had to do something. ' **She** said she went to the police, but they didn't take **her** seriously").

Importantly, whereas such non-speech-like narratives reflect the nature of the training set of GPT, and thus its limitation, written web-based narratives can be disorganized in a

manner that is equivalent to speech-like narratives. Thus, in the clinical ratings of such narratives we opted to avoid classifying this non-speech-like nature as a thought disorder per-se, only focusing on their form/organization (e.g., whether a news report is coherent or tangential and disorganized).

## Sensitivity of the simulation results to the exclusion of words repeating in two sentences/groups of N words

In the main paper, analyses of semantic distance between consecutive sentences, or consecutive groups of N words that were based on static embeddings were always preceded by removing words that appeared in both consecutive sentences (or groups of N words). Here we repeat these analyses without removing such duplicate words. This sensitivity analysis shows very consistent results (compare Figure S4 to Figure 4, and Figure S5 to Figure 5). The main difference concerned the word mover's distance measures which showed much stronger effects here.

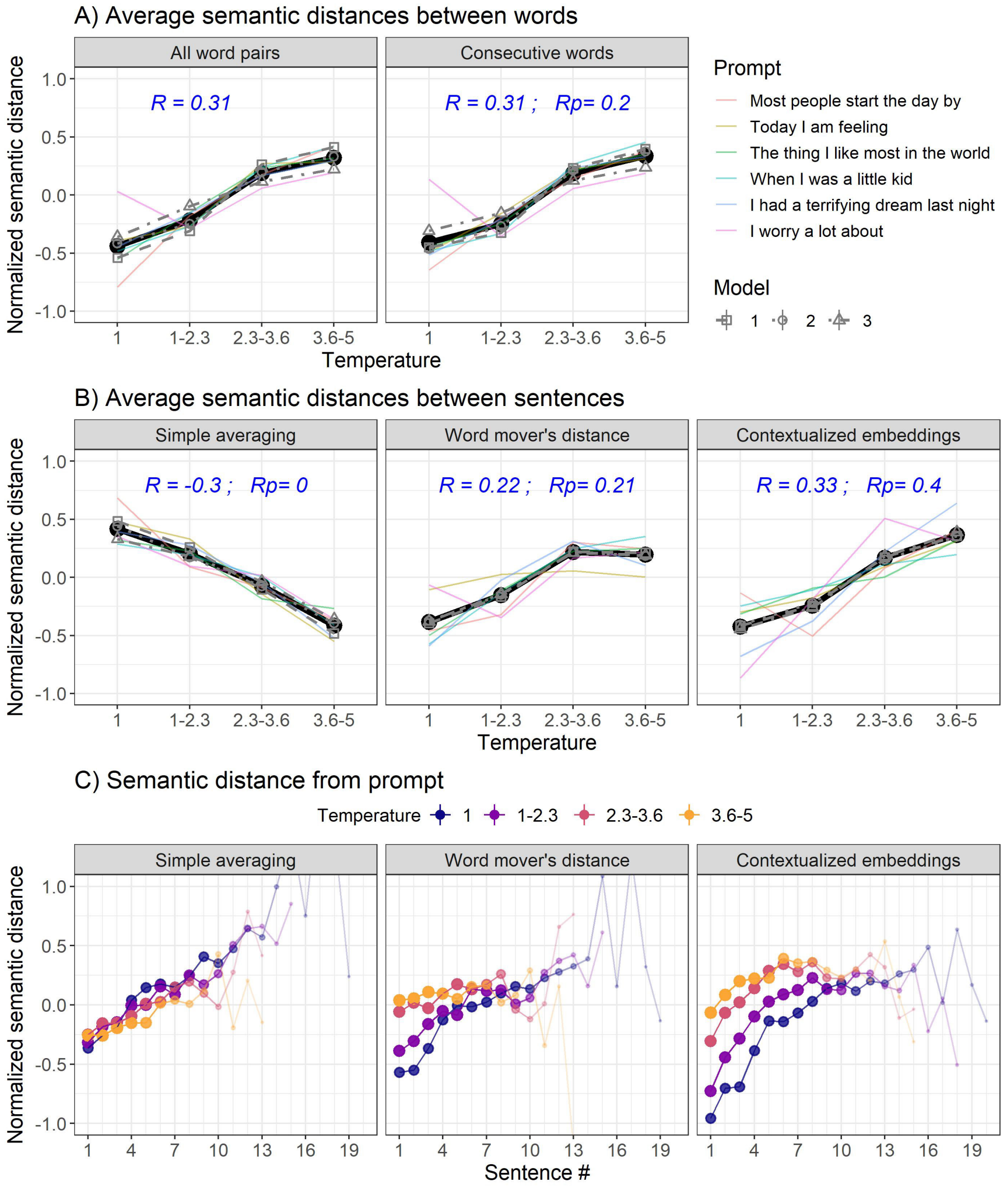


*Figure S4 - Effects of manipulating temperature on semantic distance measures of derailment (A and B) and tangentiality (C). This plot is different from Figure 3 only in measures of sentence distances using static embeddings. In the paper these measures always excluded repeating words, and here are they are included. See the caption of Figure 3 for further details.*

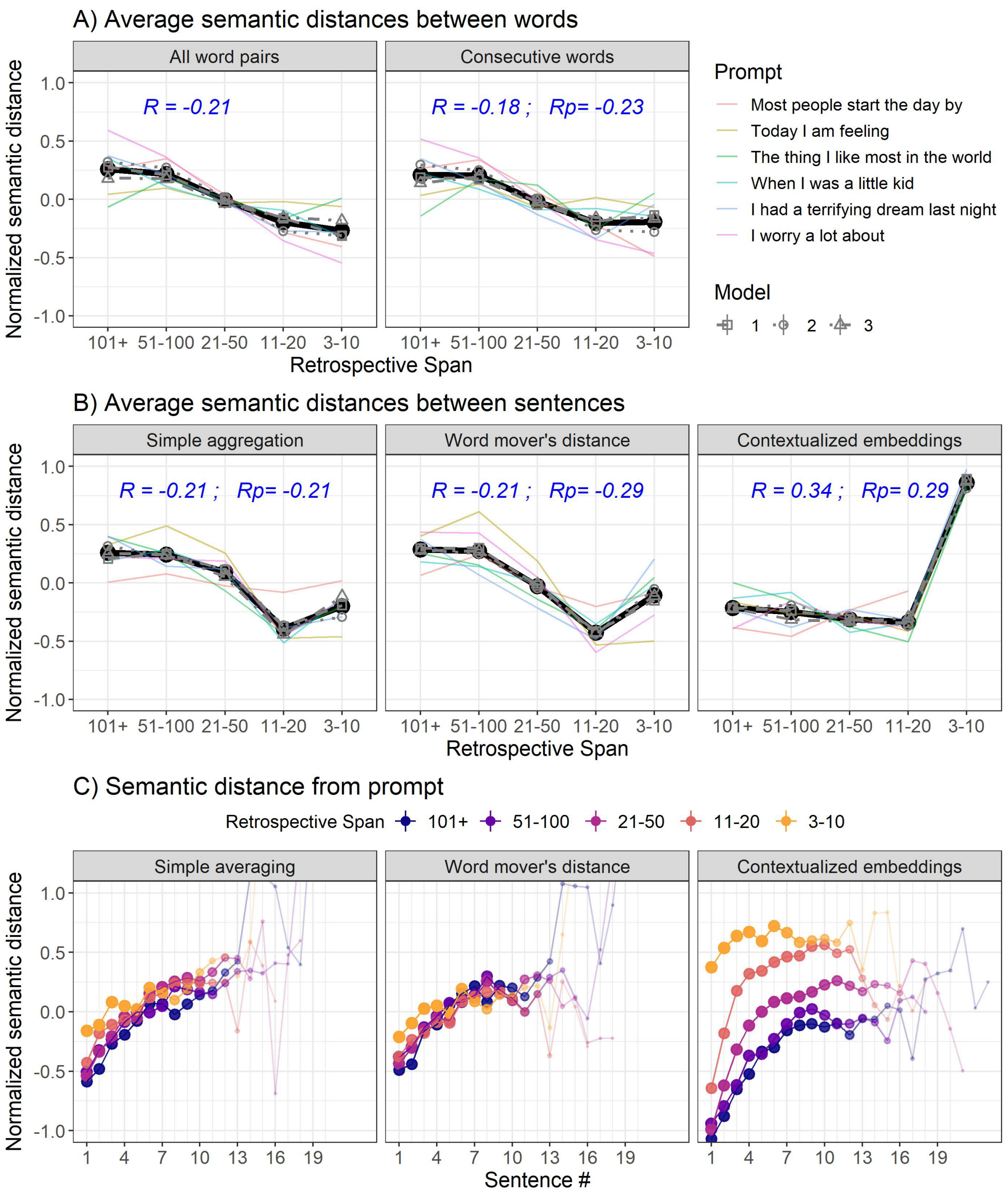


*Figure S5 - Effects of manipulating retrospective span on semantic distance measures of derailment (A and B) and tangentiality (C). This plot is different from Figure 3 only in measures of sentence or N-word group distances using static embeddings. In the paper these measures always excluded repeating words, and here are they are included. See the caption of Figure 3 for further details.*

**The effect of temperature on sentence length.**

One of our secondary findings was that higher temperature produces longer sentences. There are two main reasons for this. First, since end-of-sentence punctuation (e.g., full stops) are encoded as tokens in the model, a shallower probability distribution (caused by higher temperature) also decreases the probability that an end-of-sentence token will be generated even when it should be very likely. The second reason is that incoherent groups of words reduce the baseline probability for a sentence-ending token, because a sentence is, by definition, a group of words conveying a coherent idea. To test this second reason, we entered all generated narratives to a standard GPT-2 model (with no increased temperature), and examined the model-derived probabilities for a full-stop to be generated at each time-point (i.e., instead of each word). The results, depicted in Figure S4 confirm the role of the second reason for the original correlation between higher temperature and sentence length.

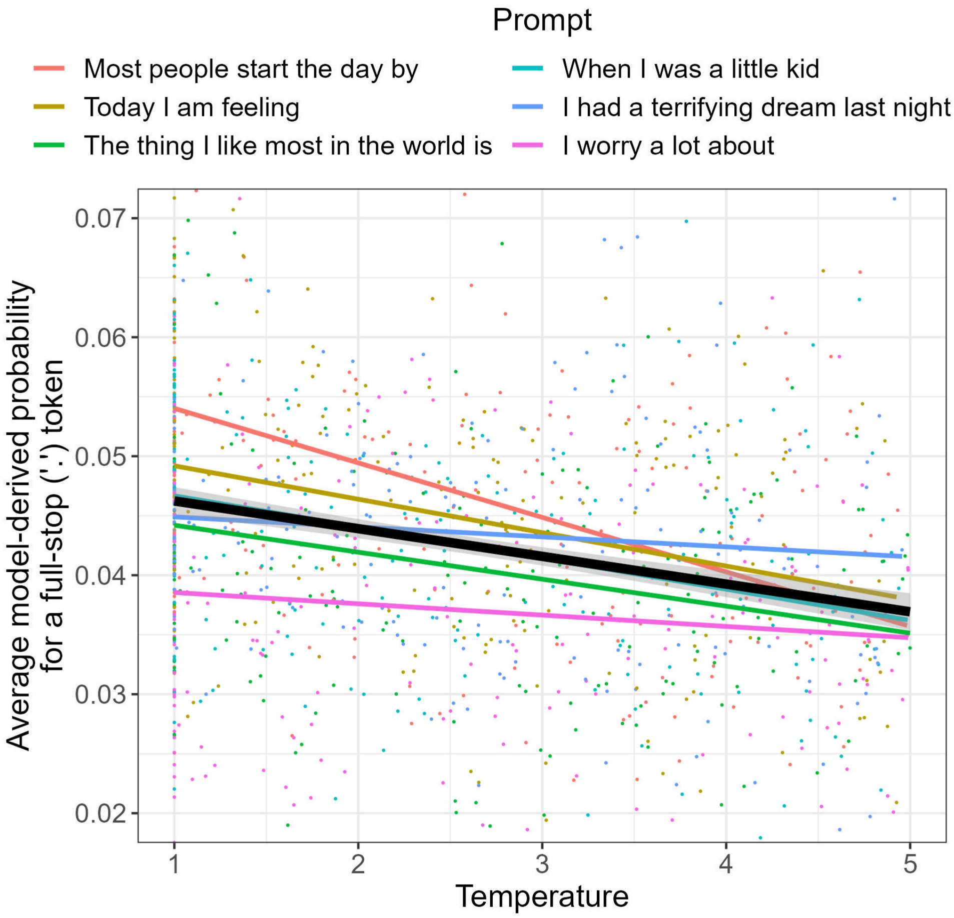


*Figure S6 - Effects of temperature on the average probability (across all tokens in each narrative) that a full-stop ('.') token will be selected.*

## Additional demonstrations of tangentiality effects

In the main paper, tangentiality was illustrated in plots wherein the relevant text-generation parameter affected the distance of the first sentence from the prompt (i.e., intercept), which made it more difficult to examine how these parameters affected the slope. Figure S7 removed intercept differences by subtracting the distance of the first sentence from all other distances. The results depicted here further strengthen the results reported in the paper, wherein only contextual memory impairment (i.e., reduced retrospective span) increased slope, and only in the transition from a span of 51-100 to a span of 21-50. Figure S8 depicts the Spearman correlations between sentence number and the sematic distance between that sentence and the prompt (while selecting only a subset of sentences to account for the non-linear effect seen in Figure S7)

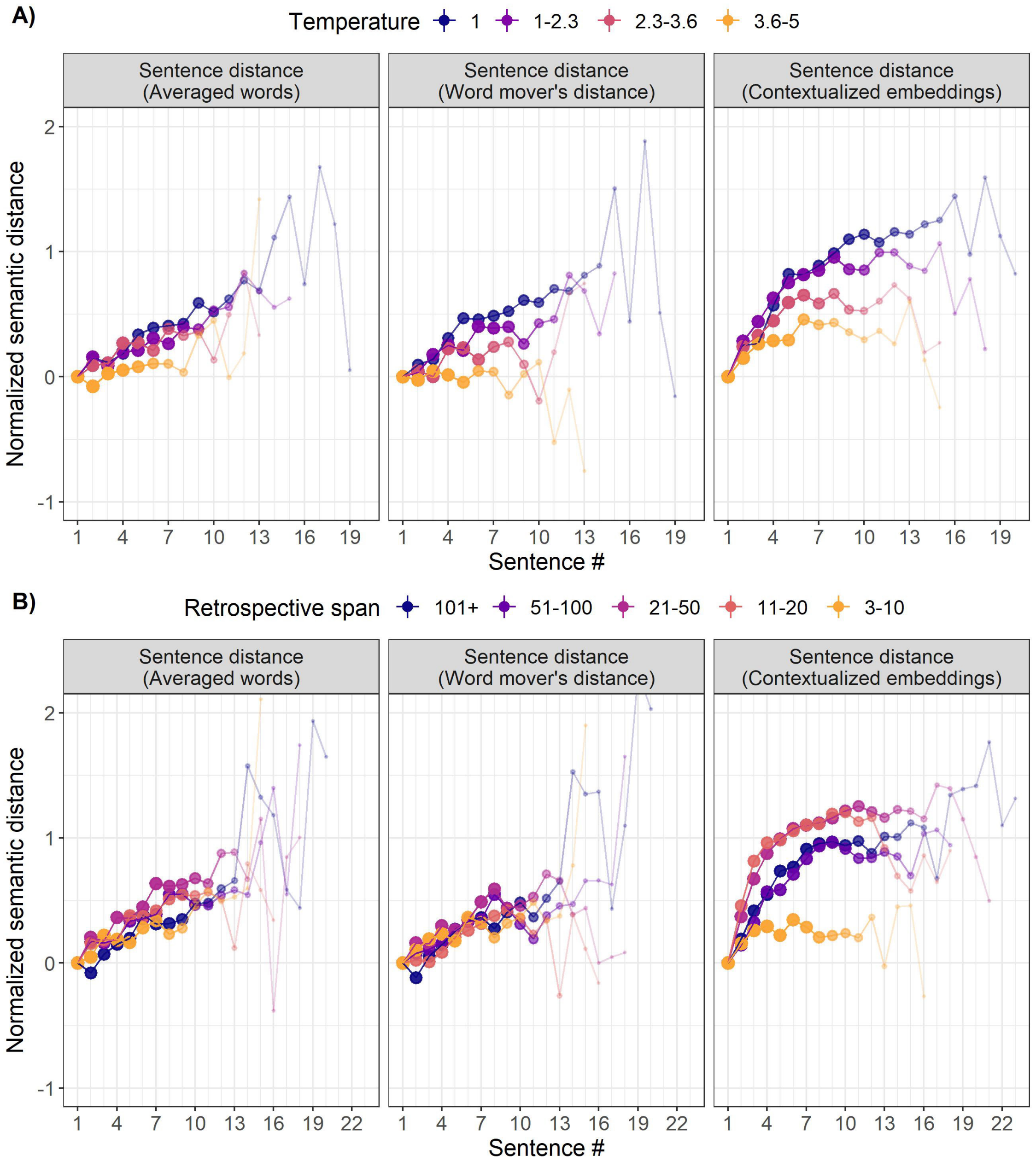


*Figure S7 – The effects of temperature (A) and retrospective span (B) on tangentiality, after removing differences in intercepts (i.e., distances of the first sentence).*

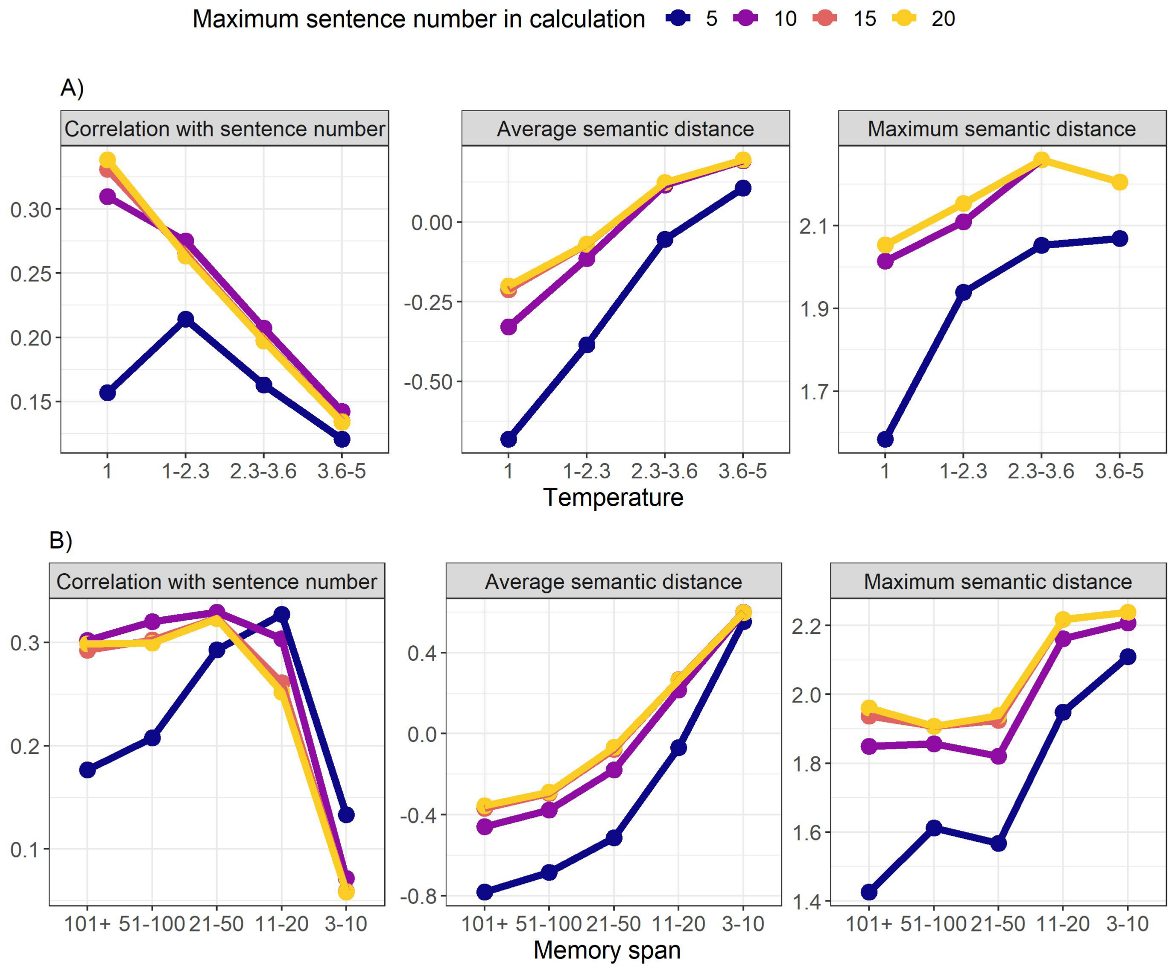


*Figure S8 - The effects of temperature (A) and memory span (B) on different measures of semantic distance from the prompt. The classic NLP measure of tangentiality focuses on increases in semantic distance over the progression of the narrative, is measured here as the Spearman correlation between sentence number and the semantic distance of this sentence to the prompt (leftmost plots). Reducing memory span, but not temperature, leads to a consistently faster loss of semantic relationship with the prompt (note that since the increase in semantic distance tended to plateau after 5-10 sentences this effect is most evident when focusing only on the first 5 sentences of each narrative, here colored in blue). We also show how aggregating semantic distance from the prompt by taking either averaging across sentences, or taking the maximally distant sentence produce similar effects for both manipulations, reflecting derailment which is equivalently measured by distanced between consecutive sentence*

## An in-depth analysis of the effects of probe type

As noted in the main text, the validity of some NLP measures varied between prompts. We visualize this variability in Figure S9 for the two most sensitive metrics (i.e., semantic distance between consecutive words, and semantic distance between consecutive contextualized sentence embeddings). The most notable finding is that variability across prompts is also moderated by text-generation parameter, and metric. Thus, we find no evidence that specific prompts or prompt types (e.g., negative vs. neural) are consistently advantageous. Rather, specific prompts activate specific semantic 'regions' that can differentially constrain the effects of increased stochasticity and limited contextual memory.

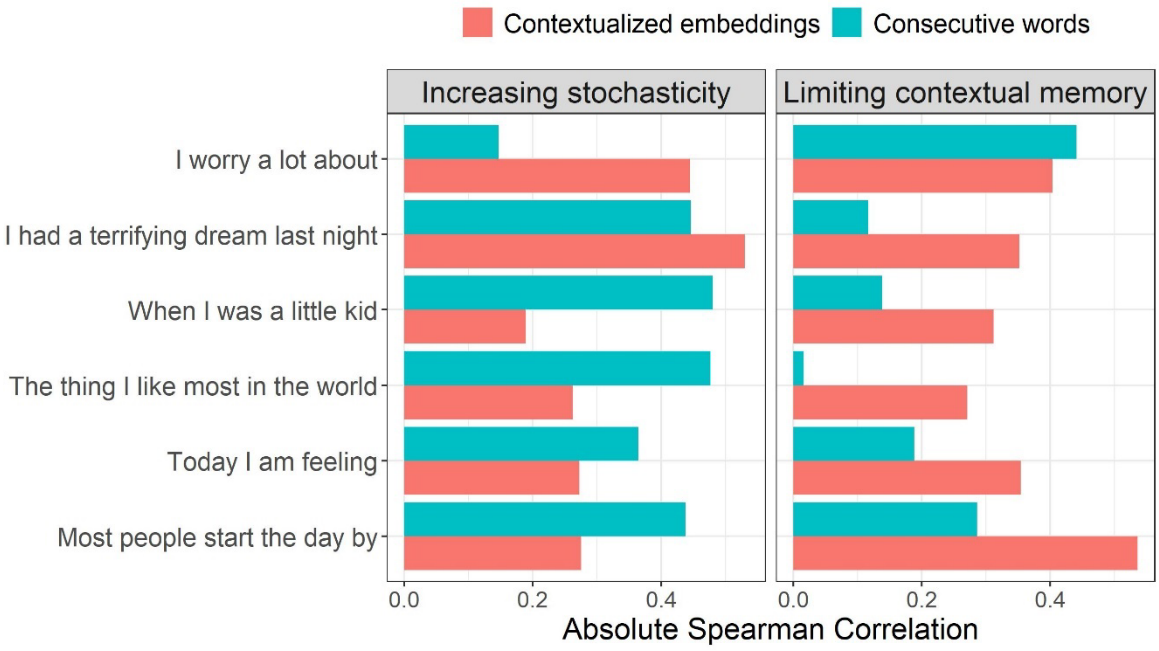


*Figure S9 – a direct comparison of effect sizes across different prompts.*

## The combined effect of increased temperature and limited memory span

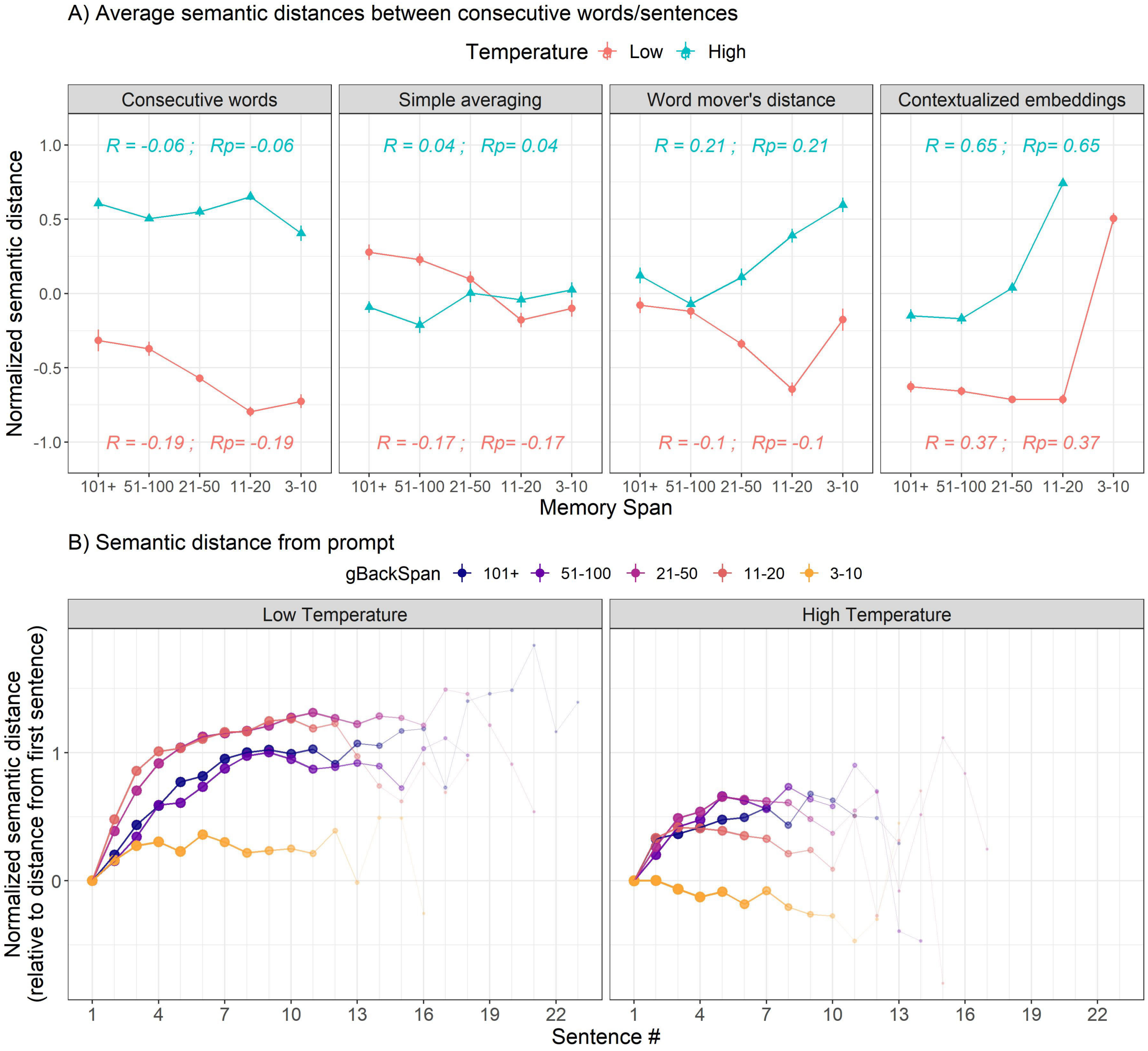


*Figure S10 - Effects of manipulating retrospective span on semantic distance measures of derailment (A and B) and tangentiality (C), under high (4) vs. low temperature (1, which reproduces the findings reported in Figure 3 in the main text). The plot clearly shows that an additional impairment formalized here as increased temperature weakens the effect of memory span on reducing semantic distance between consecutive words, and masks the tangentiality effect. Conversely, such a combined impairment amplifies the effect of memory span on semantic distance between sentences.*

**Supplementary References**


1. Corcoran CM, Cecchi GA (2020): Using language processing and speech analysis for the identification of psychosis and other disorders. *Biol. Psychiatry Cogn. Neurosci. Neuroimaging*. 5(8): 770–79.

2. Palaniyappan L, Homan P, Alonso-Sanchez MF (2022): Language network dysfunction and formal thought disorder in schizophrenia. *Schizophr. Bull.*

3. Haas SS, Doucet GE, Garg S, Herrera SN, Sarac C, Bilgrami ZR, *et al.* (2020): Linking language features to clinical symptoms and multimodal imaging in individuals at clinical high risk for psychosis. *Eur. Psychiatry*. 63(1): e72.

4. Morgan SE, Diederen K, Vértes PE, Ip SHY, Wang B, Thompson B, *et al.* (2021): Natural language processing markers in first episode psychosis and people at clinical high-risk. *Transl. Psychiatry*. 11(1): 630.

5. Just SA, Haegert E, Kořánová N, Bröcker A-L, Nenchev I, Funcke J, *et al.* (2020): Modeling incoherent discourse in non-affective psychosis. *Front. Psychiatry*. 11: 846.

6. Tang SX, Kriz R, Cho S, Park SJ, Harowitz J, Gur RE, *et al.* (2021): Natural language processing methods are sensitive to sub-clinical linguistic differences in schizophrenia spectrum disorders. *NPJ Schizophr.* 7(1): 25.

7. Voppel AE, de Boer JN, Brederoo SG, Schnack HG, Sommer I (2021): Quantified language connectedness in schizophrenia-spectrum disorders. *Psychiatry Res.* 304: 114130.

8. Voppel AE, de Boer JN, Brederoo SG, Schnack HG, Sommer IEC (2022): Semantic and acoustic markers in schizophrenia-spectrum disorders; a combinatory machine learning approach. *Schizophr. Bull.*

9. Alonso-Sánchez MF, Limongi R, Gati J, Palaniyappan L (2022): Language network self-inhibition and semantic similarity in first-episode schizophrenia: a computational-linguistic and effective connectivity approach. *Schizophr. Res.*

10. Alonso-Sánchez MF, Ford SD, MacKinley M, Silva A, Limongi R, Palaniyappan L (2022): Progressive changes in descriptive discourse in first episode schizophrenia: a longitudinal computational semantics study. *Schizophrenia (Heidelb)*. 8(1): 36.

11. Elvevåg B, Foltz PW, Weinberger DR, Goldberg TE (2007): Quantifying incoherence in speech: an automated methodology and novel application to schizophrenia. *Schizophr. Res.* 93(1–3): 304–16.

12. Rezaii N, Walker E, Wolff P (2019): A machine learning approach to predicting psychosis using semantic density and latent content analysis. *NPJ Schizophr.* 5(1): 9.

13. Corcoran CM, Carrillo F, Fernández-Slezak D, Bedi G, Klim C, Javitt DC, *et al.* (2018): Prediction of psychosis across protocols and risk cohorts using automated language analysis. *World Psychiatry*. 17(1): 67–75.

14. Bedi G, Carrillo F, Cecchi GA, Slezak DF, Sigman M, Mota NB, *et al.* (2015): Automated analysis of free speech predicts psychosis onset in high-risk youths. *NPJ Schizophr.* 1: 15030.

15. Bilgrami ZR, Sarac C, Srivastava A, Herrera SN, Azis M, Haas SS, *et al.* (2022): Construct validity for computational linguistic metrics in individuals at clinical risk for psychosis: associations with clinical ratings. *Schizophr. Res.* 245: 90–96.

16. Liebenthal E, Ennis M, Rahimi-Eichi H, Lin E, Chung Y, Baker JT (2022): Linguistic and non-linguistic markers of disorganization in psychotic illness. *Schizophr. Res.*

17. Tang SX, Cong Y, Nikzad AH, Mehta A, Cho S, Hänsel K, *et al.* (2022): Clinical and computational speech measures are associated with social cognition in schizophrenia spectrum disorders. *Schizophr. Res.*

18. Kircher T, Krug A, Stratmann M, Ghazi S, Schales C, Frauenheim M, *et al.* (2014): A rating scale for the assessment of objective and subjective formal thought and language disorder (tald). *Schizophr. Res.* 160(1–3): 216–21.

19. Andreasen NC (1986): Scale for the assessment of thought, language, and communication (tlc). *Schizophr. Bull.* 12(3): 473–82.

20. Andreou C, Bozikas VP, Papouliakos I, Kosmidis MH, Garyfallos G, Karavatos A, *et al.* (2008): Factor structure of the greek translation of the scale for the assessment of thought, language and communication. *Aust. N. Z. J. Psychiatry*. 42(7): 636–42.
